# Advances and recent trends in plant-based materials and edible films: a mini-review

**DOI:** 10.3389/fchem.2024.1441650

**Published:** 2024-08-21

**Authors:** David B. Olawade, Ojima Z. Wada, Abimbola O. Ige

**Affiliations:** ^1^ Department of Allied and Public Health, School of Health, Sport and Bioscience, University of East London, London, United Kingdom; ^2^ Department of Public Health, York St John University, London, United Kingdom; ^3^ Division of Sustainable Development, College of Science and Engineering, Qatar Foundation, Hamad Bin Khalifa University, Doha, Qatar; ^4^ Department of Chemistry, Faculty of Science, University of Ibadan, Ibadan, Nigeria

**Keywords:** plant-based materials, edible films, food packaging, plastic waste, nanocomposite

## Abstract

Plant-based materials and edible films have emerged as promising alternatives to conventional packaging materials, offering sustainable and environmentally friendly solutions. This mini-review highlights the significance of plant-based materials derived from polysaccharides, proteins, and lipids, showcasing their renewable and biodegradable nature. The properties of edible films, including mechanical strength, barrier properties, optical characteristics, thermal stability, and shelf-life extension, are explored, showcasing their suitability for food packaging and other applications. Moreover, the application of 3D printing technology allows for customized designs and complex geometries, paving the way for personalized nutrition. Functionalization strategies, such as active and intelligent packaging, incorporation of bioactive compounds, and antimicrobial properties, are also discussed, offering additional functionalities and benefits. Challenges and future directions are identified, emphasizing the importance of sustainability, scalability, regulation, and performance optimization. The potential impact of plant-based materials and edible films is highlighted, ranging from reducing reliance on fossil fuels to mitigating plastic waste and promoting a circular economy. In conclusion, plant-based materials and edible films hold great potential in revolutionizing the packaging industry, offering sustainable alternatives to conventional materials. Embracing these innovations will contribute to reducing plastic waste, promoting a circular economy, and creating a sustainable and resilient planet.

## 1 Introduction

The use of plant-based materials and edible films can be traced back several decades. In the early 20th century, natural polymers, such as cellulose, were first recognized for their potential in packaging applications. However, it was not until the 1970s and 1980s that significant advancements were made in the development of plant-based materials and edible films (Teixeira-Costa and Andrade, 2021). During this period, researchers began exploring the use of various polysaccharides, proteins, and lipids derived from plants to create films with desirable properties for packaging purposes (Zubair et al., 2022).

Polysaccharides such as cellulose, starch, chitosan, and alginate gained attention due to their abundance, low cost, biodegradability, and film-forming abilities (Cazon et al., 2017). Proteins such as soy protein and wheat gluten were also investigated for their film-forming properties and potential applications in food packaging (Fu et al., 2022; Hadidi et al., 2022). Advancements in processing techniques, such as casting, compression molding, and extrusion, allowed for the production of plant-based films with improved mechanical and barrier properties (Hadidi et al., 2022). These films exhibited promising characteristics, including biocompatibility, flexibility, and resistance to moisture and oxygen (Wang et al., 2021).

In recent years, the focus on plant-based materials and edible films has intensified due to increased awareness of sustainability and environmental issues. Researchers and industry stakeholders have been actively exploring innovative approaches to enhance the properties and applications of plant-based materials, as well as to develop edible films with improved functionality.

There has been a growing global concern regarding the environmental impact of traditional packaging materials, such as plastics, which are non-biodegradable and contribute to pollution and waste accumulation (Kumar et al., 2021). This concern has led to a significant shift towards the development and utilization of sustainable packaging materials. Sustainable packaging materials aim to minimize environmental harm by utilizing renewable resources, reducing carbon footprint, and promoting biodegradability (Rai et al., 2021).

Among the various types of sustainable packaging materials, plant-based materials and edible films have emerged as promising alternatives. Plant-based materials are derived from renewable resources such as crops, fruits, and vegetables, making them environmentally friendly (Zuin et al., 2018). Edible films, on the other hand, are thin, edible layers that can be applied to food surfaces, providing an additional protective barrier while also being safe for consumption (Falguera et al., 2011).

**FIGURE 1:**
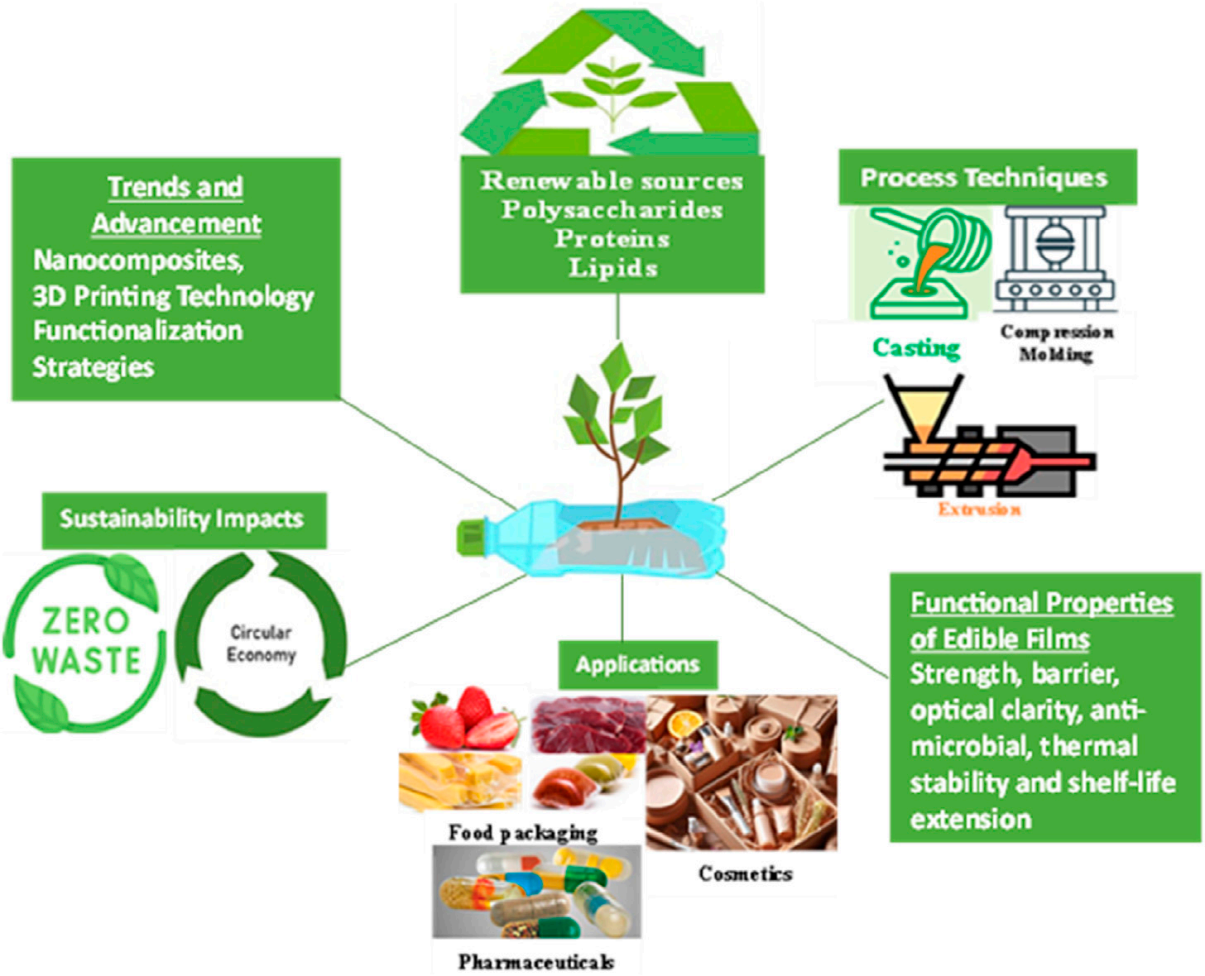
overview of plant-based materials and edible films.

By examining the advances and recent trends in plant-based materials and edible films, this comprehensive review provides valuable insights into the current state of research in this field. It highlights the potential of plant-based materials as sustainable alternatives to synthetic packaging and outlines the various production methods, properties, applications, and emerging technologies associated with edible films. The review also addresses the challenges and future directions, offering a roadmap for further research and development in this rapidly evolving area. The next sections of this review paper will delve into the advancements and recent trends in plant-based materials and edible films, including the production methods, properties, applications, and emerging technologies. Figure 1 below shows the overview of recent trends and advances in plant-based materials.

## 2 Plant-based materials vs. traditional petrochemical-based materials

The widespread use of petrochemical-based plastics, particularly in packaging applications, is a significant contributor to environmental pollution. In the mid-1900s, only about 2 million tonnes were produced globally, but as of today, over 450 million tonnes are produced yearly (Ritchie et al., 2023). This is primarily due to its cheap production cost and versatility (flexible and lightweight), making it functional for industrial applications for a host of goods and services (Zimmermann et al., 2020). Its entire lifecycle (manufacturing, utilization, and disposal) contributes just under 2 billion tonnes of carbon emissions yearly (Waste and Resources Action Programme, 2024). In addition to this, around 42% of all petrochemical-based plastics used end up in landfills, and barely a 10th is recycled (Zhao et al., 2020; Lin et al., 2023). In global economic powers like the United States and China, around 60%–85% of plastic waste generated in municipalities is sent to landfills (Lin et al., 2023). The extensive production, utilization, and improper disposal of this form of plastic have resulted in global microplastic pollution, destruction of the aquatic ecosystem, and suspected morbidity (infertility and endocrine disruption) among humans (Daltry et al., 2021; Ullah et al., 2022; Chamanee et al., 2023). They are also potential sources of harmful chemicals like per- and poly-fluoroalkyl substances (PFAS) in the environment (Sangkham, 2024).

On the other hand, plant-based biofilms seem to offer more beneficial purposes, particularly in food packaging. For example, polysaccharide biofilms like alginate have been reported to enhance the shelf life of meat and meat products by regulating permeability and restricting dehydration (Kim et al., 2018). Chitosan biofilms have preserved food quality and extended shelf life by effectively limiting oxygen and carbon dioxide transfer through the coating (Casariego et al., 2009). Others have been reported to improve food shelf life via antimicrobial properties and by increasing oxidation stability (Heydari et al., 2015; Chang et al., 2023). In addition, biopolymers have also been employed to produce antimicrobial edible coatings. In one study, soft cheese covered with biofilm prepared using chitosan embedded with antimicrobial microcrystalline cellulose and probiotic organisms for 45 days had a high antimicrobial effect against a wide range of organisms (El-Sayed et al., 2021). The stability and sensory attributes of the cheese were also reasonably maintained throughout the duration (El-Sayed et al., 2021). However, such biopolymers face limitations in structural integrity. In comparison to petrochemical-based plastics, they have reduced impact strength, lower tensile elongation, and an increased flexural modulus (Haider et al., 2019; Filho et al., 2022). For example, Young’s modulus, tensile strength, and elongation at break for the fossil-based PP are 1,100–1,500 MPa, 31–43 MPa, and 500%–650%, while respective values for gelatin are 22 MPa, 9 MPa, and 45% (Nilsen-Nygaard et al., 2021). In addition, the water vapor transmission rate for PP was reported to be between 0.1 and 0.3, while that of cellulose acetate was reportedly 257.8 (Nilsen-Nygaard et al., 2021). Plant-based biofilms also have significantly higher permeation and water absorption characteristics (Gupta P. et al., 2022). This significantly limits the application of plant-based biofilms. Some polysaccharides (amylose starch and chitosan), however, have proven to possess comparative gas (oxygen and carbon dioxide) barrier properties and tensile strength values when compared to high-density polyethene films (Cazon et al., 2017). Thus, future research aims to explore possible modifications that can be made to improve the mechanical properties of plant-based biopolymers.

Considering the environmental implications of both plastic sources, Life cycle assessments (LCAs) indicate that bioplastics generally consume fewer non-renewable resources and emit fewer greenhouse gases compared to conventional plastics, particularly when biomass energy is utilized in their production (Kurdikar et al., 2000). However, the environmental benefits of bioplastics are not without caveats. The intensive agriculture required for bioplastic production, involving the use of pesticides, herbicides, and fertilizers, can negatively impact ecosystem quality, leading to issues such as acidification and eutrophication (Gironi and Piemonte, 2011). Despite these concerns, the overall benefits of plant-based materials in food packaging are substantial. They enhance food shelf life and prevent spoilage, reducing food waste and associated environmental impacts. Additionally, plant-based biofilms help prevent the proliferation of microplastics and reduce landfill burdens due to their compostable nature (Zhao et al., 2020). In contrast, the majority of petrochemical-based plastics end up in landfills and litter in the environment, where they persist for hundreds of years, exacerbating waste management issues and contributing to environmental degradation (Lin et al., 2023; Trinh et al., 2023). However, with a wide spectrum of biofilms emerging, more LCA studies need to be conducted to determine their precise environmental impact and compare them with conventional processes (Versino et al., 2023). Table 1 provides a comprehensive comparison between plant-based and traditional petrochemical-based packaging materials.

**TABLE 1:** Plant-based vs. traditional petrochemical-based plastic packaging materials.

Property	Traditional plastic	Plant-based
Source	Petroleum (non-renewable)	Plants (renewable)
Biodegradability	Non-biodegradable	Biodegradable
Mechanical strength	High tensile strength and durability	Comparatively lower tensile strength and durability
Thermal stability	High thermal resistance	Comparatively lower thermal resistance
Cost	Established production, lower cost	Higher cost of production
Customization (3D printing)	Limited	Highly customizable
Recyclability	Limited, energy intensive (non-compostable)	Readily recyclable (compostable)
Additional functionalities	Limited	Bioactive and antimicrobial modifications possible

## 3 Plant-based materials

### 3.1 Polysaccharides

Polysaccharides are abundant carbohydrates found in plants and offer a wide range of properties that make them suitable for the development of plant-based materials and edible films. Some of the notable polysaccharides used in this context include cellulose and its derivatives, starch, chitosan, and alginate. Cellulose is the most abundant biopolymer on Earth and is derived from plant cell walls. It possesses excellent mechanical strength, thermal stability, and barrier properties (Liu et al., 2021). However, its insolubility in water limits its direct use in film formation (Xu et al., 2016). To overcome this, cellulose derivatives such as methylcellulose, carboxymethylcellulose, and hydroxypropyl cellulose are often employed (Acharya et al., 2021). These derivatives exhibit improved solubility and film-forming capabilities while retaining some of the cellulose’s desirable properties. Recent studies have also reported that acetylation of polysaccharides could improve their solubility properties (Yilmaz-Turan et al., 2020). In one study, acetylated xylan from sugarcane bagasse had significantly higher water vapor barrier and oil-resistant properties (Martins et al., 2024). Acetylation was performed using starch as blends and glycerol as a plasticizer at varying degrees of substitution (Martins et al., 2024).

Starch is another widely available polysaccharide obtained from various plant sources, primarily corn, potato, and wheat, and is highly favored due to its natural abundance and low cost (Matheus et al., 2023). It offers film-forming properties, biodegradability, and good oxygen barrier capabilities (Cheng et al., 2021). Starch-based materials can be modified through processes like plasticization, cross-linking, or blending with other polymers to enhance their mechanical strength, water resistance, and stability (Amaraweera et al., 2021). Additionally, the use of nanotechnology has enabled the development of nanocomposite films with improved properties.

Chitosan is derived from chitin, a polysaccharide found in the exoskeletons of crustaceans and cell walls of fungi. It possesses antimicrobial properties, good film-forming ability, and biodegradability (Priyadarshi and Rhim, 2020). Chitosan-based films have shown promise in various applications, including food packaging, due to their ability to extend the shelf life of perishable products by inhibiting microbial growth and maintaining product quality (Khubiev et al., 2023). Alginate is extracted from brown seaweeds and has been extensively studied for its film-forming properties. Alginate-based films are highly biodegradable, possess good water vapor barrier properties, and can be tailored to exhibit controlled release characteristics (Khorrami et al., 2021). These films find applications in food packaging, wound dressing, and pharmaceutical delivery systems. A specific example of such an application was in a recent study that developed an edible bioplastic film with increased strength and toughness by crosslinking composite films (soybean protein isolate and chitosan) with citric acid (Zhang et al., 2023). This packaging resulted in promising results like delaying fruit ripeness by about 5 days, maintaining the edible rate of cherries and strawberries by 75% 5–7 days post storage, and preventing meat spoilage via chemical inhibition and antibacterial properties (Zhang et al., 2023).

### 3.2 Proteins

Proteins derived from plant sources have gained attention as potential alternatives for plant-based materials and edible films. Soy protein, wheat gluten, casein, and other plant-based proteins are commonly explored in this regard. Soy protein isolates and concentrates are obtained from soybeans and offer a range of functional properties suitable for film formation. Soy protein-based films possess good mechanical strength, flexibility, and oxygen barrier properties (Wu et al., 2019). They can also be modified by blending with other polymers, cross-linking, or incorporating additives to enhance their properties and expand their applications. A specific example of cross-linking can be found in a recent study that co-crosslinked keratin and gluten to improve the hydrophilic characteristics of the bioplastic film, among other physicochemical properties (Alshehhi et al., 2024). Specifically, the cross-linked films had better chain mobility and higher water uptake (by about 85%) (Alshehhi et al., 2024). In another study, the incorporation of by-products of Spanish Mackerel and pectin from passion fruit resulted in bioplastic with increased strength, flexibility, biodegradability, and water vapor permeability (Florentino et al., 2022).

Wheat gluten is a byproduct of wheat processing and has received considerable attention as a sustainable film-forming material. It exhibits excellent film-forming ability, biodegradability, and gas barrier properties (Zhang et al., 2022). Wheat gluten films have been explored for food packaging applications, offering a renewable and biodegradable alternative to petroleum-based plastics. Casein is a milk protein that can form films with excellent mechanical properties, water vapor barrier properties, and transparency (Karaca et al., 2019). Casein-based films are extensively used in food packaging due to their biodegradability, good oxygen barrier capabilities, and ability to inhibit moisture transfer (Chaudhary et al., 2022). Additionally, casein films can be modified by incorporating antimicrobial agents or incorporating nanoparticles to enhance their functionality (Khan et al., 2021).

Apart from soy protein, wheat gluten, and casein, various other plant-based proteins, such as pea protein, corn zein, and hemp protein, have been explored for their potential in developing plant-based materials and edible films. These proteins offer diverse properties and functionalities, such as film-forming ability, water resistance, and mechanical strength. The development of films from these proteins involves processes such as solubilization, extraction, and film casting, which can be optimized to achieve desired film properties. A recent study has reported an industrial proof of concept by producing bioplastic films from tofu manufacturing protein-rich byproducts (Bagnani et al., 2024). Around 0.5 m^3^ of soy whey was converted into 27 kg of bioplastic films by forming protein nanofibrils with okara using methylcellulose and glycerol (Bagnani et al., 2024).

### 3.3 Lipids

Lipids derived from plant sources have also shown promise in the development of plant-based materials and edible films. Plant-based oils and fats, as well as wax-based materials, are notable examples in this category. Plant-based oils and fats, such as vegetable oils derived from soybean, corn, sunflower, or palm, can be utilized in the formulation of edible films. These films can exhibit good water vapor barrier properties, flexibility, and biodegradability (Zubair et al., 2021). For example, a study that formulated a film-forming emulsion with a hybrid of chitosan and oleic acid reported the lipid-limited transference of water vapor in the film (Aguirre-Loredo et al., 2014). By incorporating additives or modifying the lipid composition, properties such as mechanical strength and oxygen barrier performance can be improved.

Natural waxes, such as beeswax, carnauba wax, and candelilla wax, obtained from plant sources, have been explored for their potential in edible film development. These waxes offer desirable properties such as hydrophobicity, water vapor resistance, and film-forming capabilities (Saji, 2020). Wax-based films can be used as coatings on food surfaces to enhance their shelf life and preserve freshness (Singh et al., 2016). Additionally, blends of waxes with other biopolymers can be used to improve film properties. A specific example of using lipids in bioplastics for shelf-life enhancement was reported in a recent study that compounded essential oils (such as citral, carvacrol and α-terpineol) with blended films (Laorenza and Harnkarnsujarit, 2021). The addition of the essential oils improved the antimicrobial properties of the biofilm, prevented melanosis, and delayed spoilage of Pacific white shrimp (Laorenza and Harnkarnsujarit, 2021).

**FIGURE 2:**
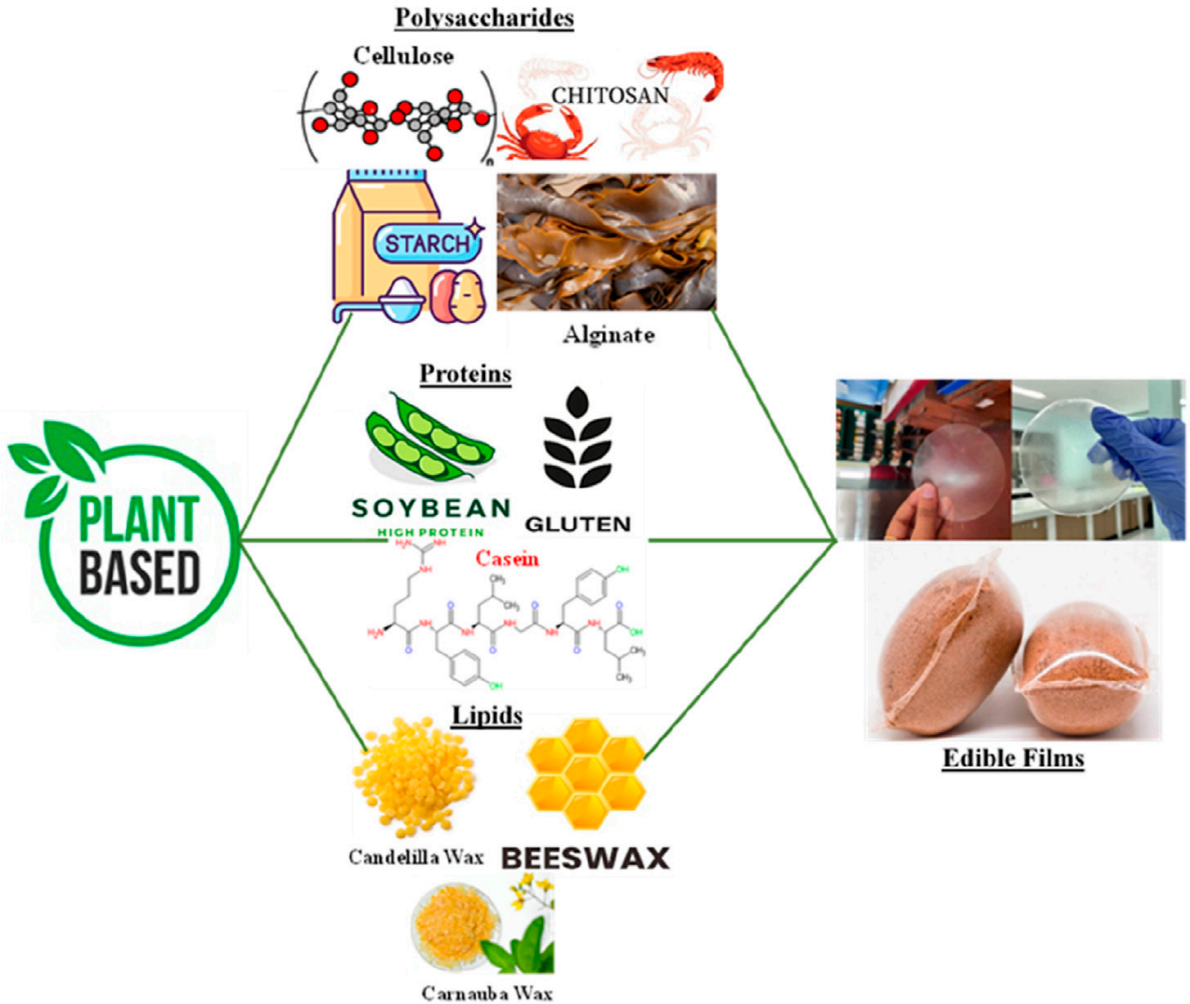
Plant-based sources for edible films.

The selection of specific plant-based materials depends on their availability, functional properties, and compatibility with the desired application. Moreover, combinations of different plant-based materials, such as blending polysaccharides with proteins or lipids, can result in composite films with enhanced properties, offering a wide range of possibilities for sustainable packaging and edible film development. Figure 2 below shows plant-based sources for edible films.

## 4 Edible films

Edible films and coatings are created from food-grade additives and biopolymers, forming thin layers that act as barriers to moisture, oxygen, and solute transfer in food products (Han, 2014; Díaz-Montes and Castro-Muñoz, 2021). These films can be used as coatings directly on food or as standalone films, gaining attention for their benefits over synthetic alternatives, such as being consumable along with the packaged product and reducing environmental pollution (Díaz-Montes and Castro-Muñoz, 2021). Recent research has concentrated on producing these films using agricultural by-products and food industry waste, integrating additives like antimicrobials, antioxidants, nutraceuticals, and flavourings to safeguard food from spoilage and pathogens, thereby improving food safety and quality (Campos et al., 2011; Díaz-Montes and Castro-Muñoz, 2021). The different types of edible films, including their applications and results, are presented in Table 2 below.

**TABLE 2:** Types, application and results of edible films.

Type of edible film	Application	Result	References
Chitosan films from shrimp shells	Extending shelf life of strawberries	Films effectively extended the shelf life of strawberries by reducing weight loss, maintaining firmness, and delaying fungal growth	Badawy et al. (2016)
Chitosan film incorporated with oregano essential oil	Extending shelf life and enhancing antimicrobial activity of chicken breast fillets	Reduced lipid oxidation, inhibited microbial growth	Zheng et al. (2023)
Soy protein isolate-based film	Extending shelf life of artichokes, eggplants and persimmons	Reduced browning and extended marketability	Ghidelli (2015)
Soy protein isolate-montmorillonite nanoclay (SPI-MMT) containing Zataria multiflora essential oil emulsion (ZEO) and nanoemulsion (ZNE)	For extending the shelf life of beef patties	Reduced microbial growth, slowed lipid oxidation, lower pH, less cooking loss, improved sensory qualities	Khosravi et al. (2023)
Chitosan Enriched with Bergamot Juice Powder Extract (CHBE)	Extending shelf life and maintaining quality of Fresh-cut fruit salad (apples, pears, kiwis, pineapples)	Even better decay reduction, highest moisture retention, better color, lowest browning and inhibited microbial growth	Demircan and Velioğlu (2024)
Carboxymethyl cellulose (CMC) - 1% concentration	Pre-storage treatment directly on mango fruits	Reduced weight loss, reduced disease incidence, delayed climacteric peak, and powered respiration rate	Ali et al. (2022)
Maltol (1%–10%) in acetylated cassava starch film	Packaging for bakery products (butter cake)	Delayed visible mold growth at room temperature an increased mold growth inhibition compared to control	Promhuad et al. (2022)
Idesia polycarpa Maxim protein composite film	Development of composite film for extending the shelf life of sweet cherries	Exhibits strong scavenging activity against various free radicals, significantly reduced weight loss and ascorbic acid loss in sweet cherries when applied as a coating and effectively extends the shelf life	Yang et al. (2023)
Chitosan (87.2% deacetylation degree)	Edible coating on tomatoes	Reduced weight loss, slower color change compared to control, reduced decrease in reducing sugar content, maintained vitamin C content, and Increased total acidity	Lukum et al. (2023)
Xanthan gum (0.5%, 1%, 2%) with 0.5 mL pomegranate peel extract	Edible coating on mangoes	Reduced weight loss, reduced respiration rate and reduced ethylene production	Kumar et al. (2022)

### 4.1 Production methods

**FIGURE 3:**
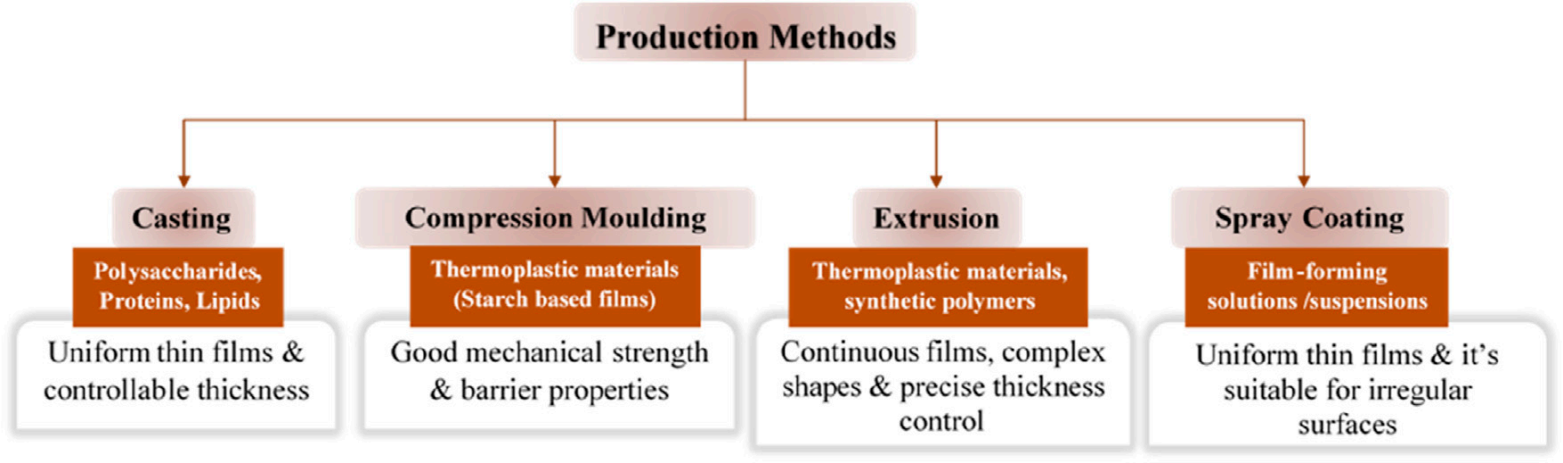
Methods for producing edible films.

Edible films can be produced using various techniques, depending on the desired properties and application requirements. Figure 3 below illustrates different methods for producing edible films, showing the relationship between the methods, materials and unique characteristics. Some commonly employed production methods include:

*Casting:* Casting involves the preparation of a film-forming solution or dispersion, which is then poured onto a flat surface or mold and allowed to dry or solidify (Siqueira et al., 2021). This method enables the formation of uniform, thin films with controllable thickness (Suhag et al., 2020). Casting is suitable for films made from polysaccharides, proteins, and lipids, as well as their combinations.

*Compression Molding:* Compression molding involves applying heat and pressure to a mixture or powder to form a solid film (Tatara, 2024). This method is commonly used for thermoplastic materials, such as starch-based films, where the material is heated and compressed in a mold to achieve the desired shape and thickness (Yu et al., 2021). Compression molding allows for the production of films with good mechanical strength and barrier properties.

*Extrusion:* Extrusion is a widely used technique for producing continuous films from melt-processed materials. The process involves melting the film-forming material and forcing it through a die to form a continuous sheet. Extrusion allows for precise control of film thickness and facilitates the production of films with complex shapes (Shim, 2013). This method is particularly suitable for thermoplastic materials, including starch, proteins, and synthetic polymers.

*Spray Coating:* Spray coating involves the deposition of a film-forming solution or suspension onto a substrate using a spray system (Suhag et al., 2020). This method allows for the formation of thin, uniform films by spraying the solution in a controlled manner. Spray coating is commonly used for the production of edible films on irregularly shaped or delicate food surfaces (Bizymis and Tzia, 2021).

### 4.2 Properties of edible films

Edible films possess a range of properties that make them suitable for various applications. The key properties of edible films include:

Mechanical Properties: The mechanical properties of edible films, such as tensile strength, flexibility, and elasticity, are crucial for their performance as packaging materials (Shah et al., 2023). These properties determine the film’s ability to withstand handling, deformation, and stress during storage and transportation.

Barrier Properties: Edible films act as a barrier against moisture, oxygen, light, and other gases, protecting the packaged product from deterioration (Kaewprachu and Rawdkuen, 2016). Barrier properties, including water vapor transmission rate (WVTR), oxygen permeability, and light transmission, are important considerations for the selection and development of edible films for specific applications (Jeevahan et al., 2020).

Optical Properties: Optical properties, such as transparency, color, and gloss, play a significant role in the visual appeal of packaged products (Kim et al., 2014). Transparent and glossy films enhance product visibility, while color additives can be incorporated to impart a desired appearance.

Thermal Properties: Thermal properties, including glass transition temperature (Tg), melting point, and thermal stability, are essential for determining the temperature resistance and processing conditions of edible films (Gounga et al., 2010). These properties impact the film’s behavior during storage, cooking, and other food processing operations.

Shelf-Life Extension Properties: Edible films can possess properties that extend the shelf life of perishable products (Kaewprachu and Rawdkuen, 2016). For example, incorporating antimicrobial agents or antioxidants into the film matrix can help inhibit microbial growth or oxidative reactions, respectively, thereby preserving product quality and freshness.

### 4.3 Applications of edible films

Edible films find diverse applications in various industries, including:

Food Packaging and Preservation: Edible films offer an eco-friendly alternative to traditional plastic packaging materials (Petkoska et al., 2021). They can be used to wrap fresh fruits and vegetables, meat, dairy products, snacks, and bakery items, providing a protective barrier against moisture loss, oxygen, and microbial contamination (Mondal et al., 2021). Edible films can also incorporate active ingredients, such as antimicrobial agents or flavoring compounds, to further enhance food safety and extend shelf life.

Biomedical and Pharmaceutical Applications: Edible films have gained attention in the biomedical and pharmaceutical fields. They can be used as drug delivery systems, where medications are encapsulated within the film matrix, allowing for controlled release and targeted delivery (Zhang et al., 2021). Edible films have also been investigated for wound healing applications, as they can provide a protective barrier while promoting moisture retention and drug release at the wound site (Borbolla-Jiménez et al., 2023). Additionally, edible films can be used for oral drug delivery, where medications are formulated into thin strips or films that dissolve in the mouth for convenient administration.

Agricultural Applications: Edible films have found applications in agriculture to improve crop quality and post-harvest preservation (Kocira et al., 2021). They can be applied as coatings on fruits and vegetables to reduce water loss, maintain firmness, and inhibit microbial growth. Edible coatings can also enhance the effectiveness of post-harvest treatments, such as controlled atmosphere storage and modified atmosphere packaging, by providing an additional protective layer (Tabassum and Khan, 2020).

Other Emerging Applications: The versatility of edible films has led to the exploration of various emerging applications. These include the use of edible films as biodegradable packaging materials for personal care products, such as soap and shampoo bars. Edible films have also been utilized as biodegradable mulch films in agriculture to promote weed control, moisture retention, and soil temperature regulation.

Overall, edible films offer a sustainable and versatile solution for packaging, preservation, and controlled delivery in various industries. Ongoing research and advancements in formulation techniques, incorporation of active ingredients, and understanding of film properties continue to expand the potential applications of edible films in the pursuit of environmentally friendly and functional packaging materials.

## 5 Recent trends and advances

### 5.1 Nanotechnology in plant-based materials and edible films

Nanotechnology has emerged as a significant area of research in the development of plant-based materials and edible films. It involves manipulating materials at the nanoscale to enhance their properties and functionalities. Some recent trends include:

Nanostructured Plant-Based Materials: Nanostructuring involves incorporating nanoparticles or nanoscale structures into plant-based materials to improve their mechanical, barrier, and antimicrobial properties (Harish et al., 2022). For example, nanocellulose, derived from cellulose, has gained attention for its exceptional strength, high surface area, and biodegradability, making it a promising nanomaterial for enhancing the properties of edible films.

Nanocomposite Edible Films: Nanocomposite edible films involve the incorporation of nanofillers, such as nanoparticles or nanoclays, into the matrix of plant-based materials to enhance their mechanical strength, barrier properties, and functional attributes (Jafarzadeh et al., 2022). These nanocomposite films exhibit improved properties, such as increased tensile strength, reduced gas permeability, and enhanced stability as shown in Table 3 below. Nanoparticles, such as silver nanoparticles, can also be incorporated into edible films to confer antimicrobial properties.

**TABLE 3:** Strategies in sustainable packaging: Advancement and challenges.

Category	Recent advances	Benefits	Challenges	Key studies
Nanotechnology	Nanotechnology in ECF Incorporation of Antimicrobial Nanoparticles (NPs) into Edible Coatings and Films (ECF)	Prolonged shelf life of fruits and vegetables Enhanced storage quality through antimicrobial properties	Electrostatic interactions affecting cell membranes. Complexities in maintaining physical and mechanical properties	Xing et al. (2019)
	Integration of Nanotechnology in Membrane Fabrication	Low energy consumption. High removal efficiency. Enhanced electromagnetic properties. Improved physicochemical stability	Balancing selectivity and productivity Incorporation of diverse nanostructures Environmental and scaling considerations	Pirayandeh et al. (2024)
	Biodegradable Polymers -Integration of Natural Polymers with Nanotechnology and Cold Plasma in Edible Films	Reduced food spoilage. Decreased pollution from synthetic plastics. Enhanced film properties through advanced technologies	Combining different polymers for optimal properties. Addressing environmental concerns. Balancing mechanical and barrier properties	Gupta P. et al. (2022)
3D Printing	Utilization of starch-based materials in 3d food printing	Customizable and precise nutrition. Ability to design unique food appearances. Simplification of the food supply chain. Effective delivery and release of active substances or drugs	Addressing varying rheological and structural requirements. Optimizing viscoelastic and film-forming properties for different applications. Integration of regulatory effects on health	Wu et al. (2023)
	Development of bio-based packaging films using conventional and additive manufacturing techniques	Potential to replace conventional petroleum-based packaging. Reduced environmental impact due to lower plastic production. Enhanced safety in food consumption. Use of biopolymer-based feedstocks in 3D printing	Limited studies on the application of 3D printing in food packaging. Need for more exploration and research. Challenges in optimizing 3D printing feedstocks and processes for food packaging	Silva et al. (2022)
Functionalization	Incorporation of organic acids, enzymes, antimicrobial proteins, phenolic compounds, probiotics, flavors, vitamins, nutraceuticals by encapsulation methods	Controls active compound diffusion. Reduces preservatives	Depends on compound type, concentration, target, encapsulation, application	Benbettaïeb et al. (2018)
	Edible active packaging systems from industry residues; antimicrobial edible coatings for fruits and meats	Increases food safety and quality. Extends shelf life. Reduces plastic and food waste Promotes circular bioeconomy	Susceptibility to harmful microorganisms. Need to maintain physicochemical properties (color, texture, moisture)	Nunes et al. (2023)

### 5.2 3D printing of plant-based edible films

Advances in 3D printing technology have opened up new possibilities for the fabrication of plant-based edible films with intricate structures and customized designs as shown in Table 3 below. Recent advancements in 3D printing have allowed for precise control over the deposition of plant-based materials, enabling the creation of complex geometries and customized shapes (Mittal et al., 2023). This technology enables the production of personalized edible films with tailored properties, such as thickness, texture, and release profiles. Additionally, the development of food-grade 3D printers and edible ink formulations has facilitated the direct printing of edible films.

While 3D printing of plant-based edible films offers exciting opportunities, challenges remain. Optimizing the rheological properties of the printing materials, ensuring their food safety and functionality, and achieving high printing resolution are ongoing areas of research. However, the potential for personalized nutrition, on-demand production, and novel sensory experiences makes 3D printing a promising technique in the field of plant-based edible films.

### 5.3 Functionalization of plant-based materials and edible films

**FIGURE 4:**
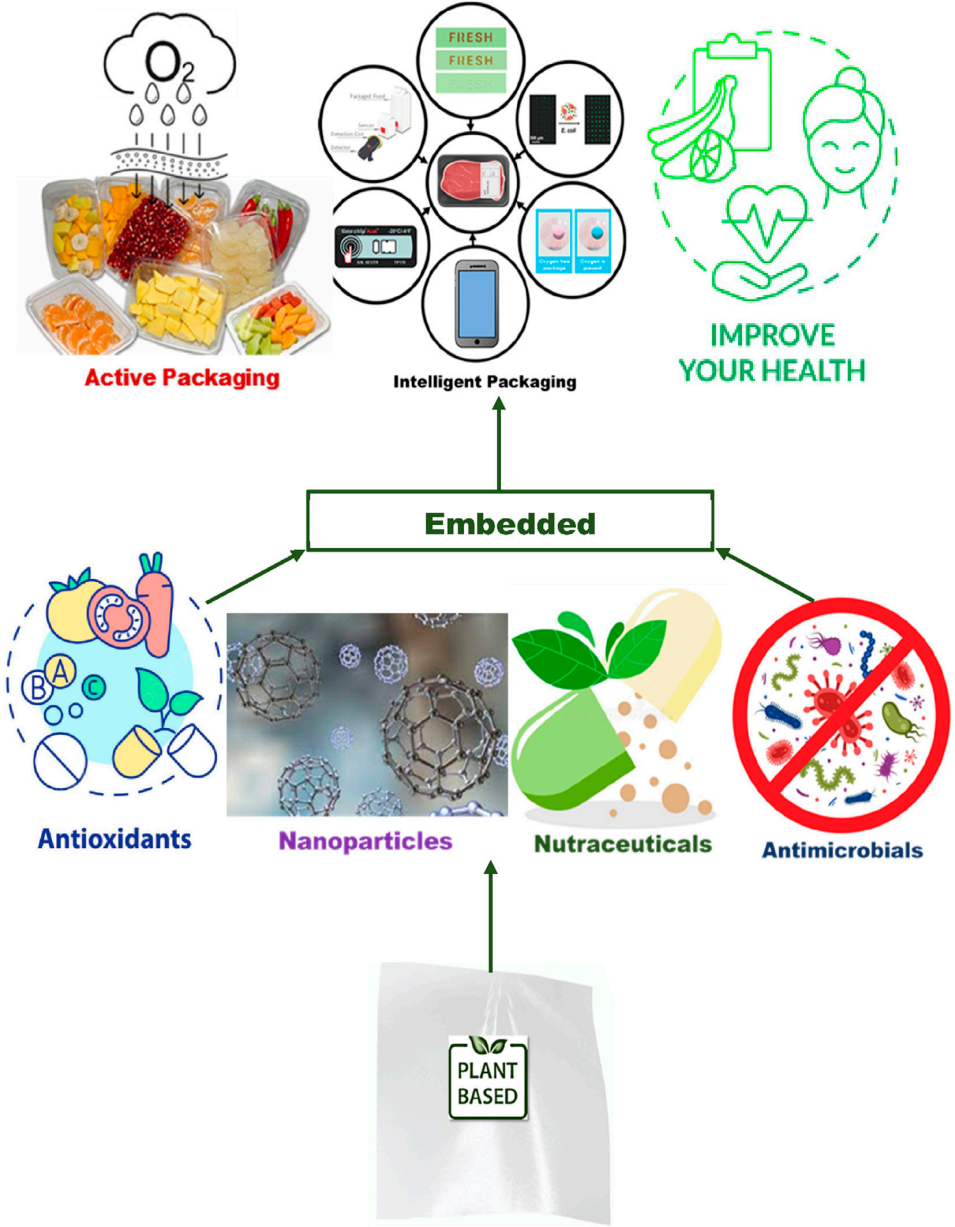
Cross-sectional view of functionalized plant based edible films.

Functionalization involves incorporating additional properties or functionalities into plant-based materials and edible films to enhance their performance and value as shown in Figure 4. Active and intelligent packaging systems involve incorporating active components, such as antimicrobial agents, oxygen scavengers, or moisture absorbers, into the packaging materials (Dobrucka and Cierpiszewski, 2014). These components can help extend the shelf life of packaged products, improve food safety, and enhance product quality. Plant-based materials and edible films can be functionalized with active agents to create active packaging systems that interact with the packaged product and its environment (Dutta and Sit, 2022).

Plant-based materials and edible films can be functionalized by incorporating bioactive compounds, such as antioxidants, antimicrobials, or nutraceuticals (Daniloski et al., 2021). These compounds can provide additional health benefits, enhance food preservation, and improve the sensory attributes of packaged products. Plant extracts, essential oils, and natural antimicrobial agents can be incorporated into the film matrix to impart bioactive properties.

The development of plant-based materials and edible films with inherent antimicrobial properties has gained significant attention (Manzoor et al., 2023). This includes the incorporation of natural antimicrobial agents or the modification of the film surface to create antibacterial or antifungal properties. These antimicrobial edible films can help prevent microbial growth, reduce the need for chemical preservatives, and enhance food safety (Punia et al., 2021).

These recent trends and advances in plant-based materials and edible films highlight the continuous efforts to enhance their properties, functionality, and application potential. The integration of nanotechnology into plant-based materials has allowed for the development of nanostructured materials and nanocomposite edible films, which exhibit improved mechanical strength, barrier properties, and antimicrobial activity (Jafarzadeh et al., 2022). These advancements open up possibilities for creating sustainable and high-performance packaging materials. The utilization of 3D printing technology in the production of plant-based edible films offers opportunities for customized designs, complex geometries, and personalized nutrition. Despite some challenges in terms of material rheology and printing resolution, 3D printing holds promise for on-demand production and novel sensory experiences.

Functionalization of plant-based materials and edible films with active and intelligent packaging components allows for the creation of packaging systems that interact with the packaged product and its environment. Incorporating bioactive compounds into these materials enhances their health benefits, food preservation capabilities, and sensory attributes (Salgado et al., 2015). Furthermore, the development of plant-based materials with inherent antimicrobial properties provides a natural alternative to conventional antimicrobial agents, reducing the need for synthetic preservatives.

These recent trends and advances in plant-based materials and edible films contribute to the overall goal of sustainable and eco-friendly packaging solutions. By harnessing nanotechnology, 3D printing, and functionalization strategies, researchers and industries are continuously exploring innovative approaches to improve the properties, functionalities, and applications of these materials. The future of plant-based materials and edible films holds immense potential in addressing the growing demand for sustainable packaging and promoting a more environmentally conscious and healthier society.

## 6 Challenges and future directions

While significant progress has been made in the development of plant-based materials and edible films, challenges related to sustainability, regulation, biodegradability, and performance optimization remain. Future directions aim to overcome these challenges through advanced processing techniques, circular economy approaches, multifunctional materials, and collaborative efforts. By addressing these challenges and advancing research in the field, plant-based materials and edible films have the potential to revolutionize the packaging industry and contribute to a more sustainable and environmentally friendly future.

One of the key challenges in the field of plant-based materials and edible films is ensuring sustainability throughout the entire lifecycle, from sourcing raw materials to disposal. The availability and scalability of plant-based resources need to be carefully considered to meet the growing demand for sustainable packaging materials. Additionally, the development of efficient and eco-friendly processing techniques is crucial to minimize energy consumption and environmental impact.

The regulatory landscape surrounding plant-based materials and edible films is evolving, and there is a need for clear guidelines and standards. It is important to ensure that these materials meet safety requirements and are compliant with relevant regulations for food contact materials. Moreover, consumer acceptance and perception play a vital role in the widespread adoption of plant-based packaging. Educating consumers about the benefits and sustainability aspects of these materials can contribute to their acceptance and market penetration.

Achieving true biodegradability and compostability is a significant challenge for plant-based materials and edible films. While many plant-based materials are biodegradable, their degradation rate and compatibility with existing waste management systems need to be considered (Moshood et al., 2022). Developing materials that can readily biodegrade in various environments, including home composting or industrial composting facilities, is crucial for reducing the environmental impact of packaging waste.

Further research and development efforts are needed to optimize the performance of plant-based materials and edible films (Gaspar and Braga, 2023). This includes improving mechanical strength, barrier properties, and stability to meet the diverse requirements of different applications. Enhancing the film’s functionality, such as incorporating active agents, improving printing resolution, and tailoring release profiles, will broaden the range of potential applications.

Future directions in the field of plant-based materials and edible films include:

1. Advanced Processing Techniques: Exploring advanced processing techniques, such as nanotechnology, electrospinning, and biofabrication, can further enhance the properties and functionalities of plant-based materials. These techniques enable precise control over material structures and properties, opening up new possibilities for tailored applications.
2. Circular Economy Approaches: Focusing on circular economy principles, such as designing materials for recyclability and developing closed-loop systems, can promote the sustainable use and disposal of plant-based packaging. Creating systems that enable efficient collection, recycling, and upcycling of plant-based materials will contribute to a more sustainable packaging industry.
3. Multifunctional Materials: Developing plant-based materials with multifunctional properties can offer added value and expand their application potential. These materials can possess not only barrier and mechanical properties but also functionalities such as antimicrobial activity, moisture management, and intelligent sensing capabilities.
4. Collaboration and Knowledge Sharing: Collaboration among researchers, industries, and regulatory bodies is essential for driving innovation, addressing challenges, and sharing knowledge in the field of plant-based materials and edible films. Open communication and interdisciplinary approaches can accelerate the development and adoption of sustainable packaging solutions.

## 7 Conclusion

The advancements in plant-based materials and edible films have the potential to revolutionize the packaging industry and promote sustainable practices. The use of renewable resources reduces reliance on fossil fuels and decreases carbon emissions. By replacing conventional plastic packaging with plant-based alternatives, we can mitigate environmental issues associated with plastic waste, such as pollution and landfill accumulation.

Furthermore, the future prospects of plant-based materials and edible films are promising. Ongoing research and development efforts are focused on addressing challenges related to sustainability, scalability, regulation, and consumer acceptance. Advancements in processing techniques, such as nanotechnology and 3D printing, offer exciting possibilities for tailoring materials and creating customized packaging solutions. Additionally, the functionalization of plant-based materials with active and intelligent components enhances food safety, preservation, and quality.

Collaboration among researchers, industries, and regulatory bodies is crucial for driving innovation and knowledge sharing in the field. This collaboration can facilitate the development of standardized guidelines and regulations for plant-based materials and edible films, ensuring their safety and compliance with food packaging requirements.

In conclusion, plant-based materials and edible films have the potential to significantly impact the packaging industry by providing sustainable alternatives to conventional materials. With ongoing research, technological advancements, and increased consumer awareness, these materials can contribute to a more environmentally friendly and sustainable future. By embracing these innovations, we can move towards a circular economy, reduce plastic waste, and create a more sustainable and healthier planet for future generations.
